# National‐Standard Middle‐Distance Runners Maintain 1500 m Time Trial Running Performance on Successive Days

**DOI:** 10.1002/ejsc.70142

**Published:** 2026-02-10

**Authors:** Laurence P. Birdsey, Steven Brown, Thomas Dos’Santos, Daniel Evans, Adam Runacres, Matthew Weston, Adam Field

**Affiliations:** ^1^ Department of Sport and Exercise Sciences Manchester Metropolitan University Institute of Sport Manchester UK; ^2^ UK Sports Institute Manchester UK; ^3^ Institute for Sport Physical Education and Health Science Moray House School of Education and Sport University of Edinburgh Edinburgh UK

**Keywords:** athletes, fatigue, recovery, repeated performance, track and field

## Abstract

To examine how middle‐distance athletes maintain self‐paced time trial performance on successive days, 12 national‐standard middle‐distance specialists performed two self‐paced 1500 m time trials on successive days. Following baseline assessment and familiarisation trials, participants (10 male, 2 female, mean age ± SD: 27 ± 7 years, mass: 66 ± 8 kg, height: 1.80 ± 0.08 m, season best 1500 m time: 243.9 ± 18.4 s) performed two 1500 m time trials separated by 24 h on an instrumented treadmill. Internal (respiratory exchange ratio; RER, oxygen uptake, blood lactate concentration, heart rate, session and differential ratings of perceived exertion) and external (speed and time) measures quantified exercise intensity. Step length and frequency were collected from integrated force transducers (1000 Hz). All variables were log transformed before analysis with mixed linear models. The uncertainty (90% confidence interval) of our between‐trial differences (trial 2 vs. trial 1) for all measures other than peak and mean RER were equivalent to previously reported measurement errors. Additionally, athletes were ∼2.5 times more likely to perceive greater exertion for time trial two, but with considerable uncertainty around the estimates. National‐standard middle‐distance specialists maintain 1500 m time trial running performance on successive days without the use of any structured recovery interventions.

## Highlights

- National‐standard middle‐distance specialists maintain 1500 m time trial running performance on successive days, without the use of any structured recovery interventions.

## 1 Introduction

Athletics championships, such as the British Athletics National Championships, require competitors in the 1500 m events to compete over consecutive days, with heats and finals performed on day one and two respectively. In this format, success depends not only on peak performance capacity, but also on the ability to recover and reproduce peak running performances within a short time frame. This challenge is unlikely to be uniform across competitors and is most pronounced for athletes who must perform closer to their individual physiological capacity to progress, rather than those able to qualify at lower relative intensities. Middle‐distance racing involves complex tactics (Casado et al. 2021), with athletes often adopting pacing strategies to maximise the potential of winning, rather than performing the fastest possible time (Casado et al. 2024; Thiel et al. 2012). Eventual medallists are frequently able to progress from earlier rounds while running below their season's or personal best in elite competitions, whereas lower‐ranked athletes may be required to operate closer to their maximum capacity simply to advance. As such, apparent differences in performance across rounds may reflect differences in the relative physiological and mechanical cost of qualification rather than absolute race time alone.

It is anecdotally suggested that some high‐performing athletes may deliberately temper their effort in early rounds. This restraint is not only driven by energetic conservation, but also by concern that repeated maximal running efforts may compromise recovery, elevate musculoskeletal strain or impair subsequent performance. However, whether such caution is physiologically necessary remains unclear. If athletes are capable of reproducing running performance across consecutive days, conservative early‐round strategies may be unnecessarily limiting and potentially increase exposure to tactical risk. Empirical evidence addressing this issue is currently lacking. Data from elite championships provide partial insight but also highlight key limitations. Analyses of men's 1500 m events across Olympic Games and World Championships indicate that mean finishing times of finalists do not differ substantially between heats, semi‐finals and finals (Hanley et al. 2019). However, this finding reflects a progressively selected athlete pool rather than constant individual performance. For instance, athletes eliminated in earlier rounds consistently record slower times, suggesting that qualification demands impose a higher relative load on those with lower performance capacity. This distinction highlights the importance of separating absolute race outcomes from the relative intensity at which performances are achieved when considering fatigue and recovery.

The consequences of maximal running may be particularly important in this context. Unlike non‐load‐bearing endurance sports such as rowing or cycling, running involves repeated eccentric muscle actions, high ground reaction forces and a flight phase, all of which are associated with greater neuromuscular disruption (Lepers et al. 2000; Millet, Lepers, et al. 2002; Millet, Martin, et al. 2002; Millet et al. 2003). These mechanical demands may exacerbate residual fatigue following maximal running and impair the ability to reproduce performance within 24 h. Consistent with this, maximal rowing ergometer performance can be maintained 24 h after a prior effort (Hopkins et al. 2011), whereas 5000 m running performance is impaired over the same recovery period following an initial maximal trial (Bosak et al. 2009). Importantly, the relevance of these findings lies not in the difference in event distance, but in the shared mechanical characteristics of maximal running tasks, which impose substantially greater impact‐related loading than non‐running endurance modalities. These contrasting findings suggest that recovery timelines derived from non‐impact sports may not translate directly to running, particularly when maximal efforts are required. The 24 h recovery window therefore represents a relatively short interval in the context of middle‐distance running competitions. While elite athletes may have access to structured recovery strategies, such approaches are not universally available or consistently implemented. Understanding whether maximal running performance and its underlying determinants can be recovered within 24 h in the absence of extensive recovery interventions has clear practical relevance. Importantly, evidence supporting the reproducibility of maximal performance may provide athletes and coaches with greater confidence to perform closer to true capacity in earlier rounds, rather than adopting conservative strategies driven by fear of residual fatigue or give confidence to maintaining tactics in subsequent rounds.

Determinants of 1500 m performance include running economy (Bellinger et al. 2021; Ingham et al. 2008), maximal oxygen uptake (Ingham et al. 2008), velocity at maximal oxygen uptake (Bellinger et al. 2021) and maximal blood lactate concentration (Ferri et al. 2012), with step frequency and length determining running speed (Hunter et al. 2004). It is therefore plausible that overall performance may be preserved despite underlying alterations in these determinants following prior maximal exertion. As performance differences could be small and difficult to detect, assessing both performance and its contributing factors may provide a more sensitive indication of residual fatigue than race time alone.

At present, there are no reports on the impact of a prior 1500 m time trial on subsequent performance in highly trained middle‐distance athletes. Simulated trials offer a standardised way to examine how time trial efforts influence next‐day performance in the absence of external tactical influences. Understanding the impact of prior 1500 m time trial performance on overall subsequent performance, along with the physiological and biomechanical determinants, may inform the design and implementation of specific training and recovery strategies in an attempt to enhance competition performance. Therefore, the purpose of this study was to examine the impact of successive 1500 m self‐paced time trials on running performance and determinants of running performance, in national‐standard middle‐distance athletes.

## 2 Materials and Methods

### 2.1 Participants

Twelve (two female) national‐standard middle‐distance athletes (mean ± standard deviation [SD]: age: 27 ± 7 years, mass: 66 ± 8 kg, height: 1.80 ± 0.08 m) from a local athletics club voluntarily completed all study procedures. All athletes specialised in middle‐distance events (800 and/or 1500 m), had the same highly experienced coach, performed a structured and periodised training programme, competed in national‐standard or international‐standard competitions, had season best (SB) times of within 20% of the world record and were classified as tier 3 or above (McKay et al. 2022). This study was conducted in November and December 2023, following a minimum six‐week training period after the athletes' off‐season break. Individualised training programmes were followed, typically involving running six times per week, including a minimum of four interval sessions performed at middle‐distance pace and resistance exercise one to two times per week. Institutional ethical approval was granted (details removed for review) before participant recruitment. Athletes were informed of the purpose and procedures of the investigation prior to providing written informed consent and completing a health screening questionnaire. All health and safety procedures were adhered to throughout the completion of this research study.

### 2.2 Design

This within‐subject observation study involved two 1500 m time trials separated by 24 h. Athletes visited the laboratory on four occasions; visit one to undertake baseline assessments, visit two to complete a familiarisation trial and visits three (time trial 1) and four (time trial 2) to complete 1500 m time trials. Visits one, two and three were separated by a minimum of three and a maximum of 10 days, whereas visits three and four were performed at the same time of day, separated by 24 h (± 1 h) to control for circadian variation. Athletes were instructed to wear the same clothing and footwear between trials, with instructions to use their preferred footwear appropriate for a short road race (i.e., lightweight racing shoes, as spikes could not be worn on the treadmill), which included the use of carbon plated shoes. Running intensity was quantified internally (heart rate [HR], expired gas analysis, session ratings of perceived exertion [sRPE], differential ratings of perceived exertion [dRPE], blood lactate concentration [BLA]) and externally (speed, time). Additionally, spatiotemporal variables were assessed for each step, including step frequency (SF) and step length (SL).

During visit one, athletes undertook sub‐maximal and maximal running tests to determine the warm‐up speeds and starting speeds for the time trials. Subsequently, athletes were permitted to run at self‐determined speeds for up to 10 min to familiarise themselves with the self‐paced nature of the treadmill. The self‐pace mode of the treadmill enables users to regulate the treadmill belt speed (i.e., the treadmill belt speed adapts to the athlete's speed), enabling a self‐paced time trial to be performed. During visit two, participants were fully familiarised with all procedures to be employed in visits three and four, including a 1500 m time trial. For visit three, a 1500 m self‐paced time trial was performed. As all athletes were highly experienced competitors with their own routines, athletes were instructed to eat and drink as if preparing for competition and recorded food intake for visit three, which was replicated for visit four. As structured recovery interventions may attenuate fatiguing responses or enhance the rate of recovery following maximal running exercise (Brophy‐Williams et al. 2017; Dupuy et al. 2018), athletes were instructed to avoid any structured recovery or recovery aids (e.g., ice baths, active recovery, compression clothing) before completing visit four, which occurred 24 h (± 1 h) later, when all measures were repeated.

### 2.3 Sub‐Maximal and Maximal Assessments

The sub‐maximal assessment was completed on a motorised and instrumented treadmill (Gaitway 3D, h/p/cosmos sports and medical GmbH, Germany), whereby athletes performed a series of four‐minute stages separated by 45 s to undertake capillary blood sampling (Biosen C‐line, EKF Diagnostics, Germany). Starting speed was individualised with the aim of at least two stages without an initial rise in blood lactate concentration and increased by 1 km·h^−1^ until a second and distinct rise in blood lactate concentration was observed. Following a 10‐min recovery period a maximal assessment was performed. This commenced 1 km·h^−1^ slower than the final speed in the sub‐maximal assessment, with speed increasing by 0.1 km·h^−1^ every 12 s (0.5 km·h^−1^ per minute) until volitional exhaustion (Hanley and Shaw 2022). A capillary blood sample was obtained immediately post and 60 s post to determine peak BLA. Lactate threshold (LT) was determined as the speed immediately preceding the first distinct rise in blood lactate concentration, with lactate turn point (LTP) the second distinct and sustained rise in blood lactate concentration (Jones and Doust 1998). These were determined visually (Jones and Doust 1998), undertaken by two independent and experienced researchers (LB, AF). Expired gas analysis (Vyntus CPX Metabolic Cart, Vyaire Medical Inc., USA) and heart rate (Polar H10, Polar Electro Oy, Helsinki, Finland) was recorded throughout. Rolling 30‐s mean values for oxygen uptake were calculated during the ramp assessments, with the highest value termed $\dot{V}$O_2_peak. As $\dot{V}$O_2_peak was not a criterion outcome of this study, a validation protocol was not employed (Poole and Jones 2017). A rolling 60‐s mean for speed during the ramp assessment was calculated and the highest value reported as velocity at $\dot{V}$O_2_peak (v$\dot{V}$O_2_peak).

### 2.4 Time Trials

Athletes reported to the laboratory at the same time of day across both trials (between the hours 0900 and 1830 h). A standardised warm‐up was performed consisting of 5 min at LT, 5 min at LT plus 50% of the difference between LT and LTP and 30 s at v$\dot{V}$O_2_peak each separated by 45 s. Athletes were then permitted to perform stretches, running drills and strides, as per their usual preparations for competition, which was recorded and replicated for the second time trial. Twenty minutes after completion of the running portion of the warm‐up athletes completed a 1500 m self‐paced time trial and were instructed to complete the time trial as quickly as possible. As the mean speed in 1500 m time trials is around 101% (Bellinger et al. 2021) to 103% (Spencer and Gastin 2001) of v$\dot{V}$O_2_peak and as athletes typically speed up during 1500 m competitions (Hanley et al. 2019), 100% of v$\dot{V}$O_2_peak was used as an appropriate and standardised, starting speed for all trials. Participants were able to vary speed throughout by changing their position on the treadmill belt (based on centre of pressure on treadmill), thereby permitting a self‐paced effort. Athletes were blinded to speed; however, they were provided distance covered on the treadmill display and were provided verbal information every 400 m (e.g., ‘3, 2 and 1 lap(s) remaining’) and for the final 200m (‘200m remaining’), without any other verbal feedback or encouragement. Time trials were conducted in temperate conditions (temperature: 20.2 ± 0.7°C, humidity: 43.5 ± 6.3%).

### 2.5 Exercise Intensity

Heart rate telemetry (recorded at 1 Hz) and expired gas analysis was conducted throughout all running exercises with data exported to Microsoft Excel for analysis. For the time trial warm‐ups, a mean of the final 60 s of the two initial speeds was calculated and for the time trials an overall mean was calculated as well as a peak, defined as the highest 30‐s rolling average. Expired gas analysis data from the two initial speeds of the warm‐up was used to assess running economy, calculated as the energy cost of running to account for changes in substrate utilisation and calculated using updated nonprotein respiratory quotient equations (Peronnet and Massicotte 1991) and energy equivalents for substrates metabolised at moderate to high speeds (Jeukendrup and Wallis 2005). Speed and distance were recorded continuously through the time trials using the manufacturer's proprietary software (Gaitway 3D version 1.9.0, h/p/cosmos sports and medical GmbH, Germany). Capillary blood samples were obtained (BLA) immediately, 1 min and every 2 min post time trial until a peak was reached and a reduction was subsequently observed.

### 2.6 Ratings of Perceived Exertion

Athletes recorded sRPE and dRPE for breathlessness (dRPE‐B) and leg‐muscle (dRPE‐L) exertion in a randomised and counterbalanced order 3 min after completing the time trial. Ratings were provided using a numerically blinded CR100 scale with verbal anchors and athletes were familiarised with these measures during the familiarisation visit. Differential ratings of perceived exertion provide a detailed quantification of internal load during team‐sport activities (McLaren et al. 2017), are sensitive to external load (Birdsey et al. 2019) and distinguish between different areas of effort (Weston et al. 2015).

### 2.7 Time Trial Ground Reaction Force

For all running trials, incline was set at 1% to replicate the metabolic demands of outdoor running (Jones and Doust 1996). Tri‐axial ground reaction force data were recorded by the instrumented treadmill at 1000 Hz and low‐pass Butterworth filtered at 30 Hz. Using the manufacturer's preset algorithms, step length and frequency were determined for each step and a mean calculated for each time trial.

### 2.8 Statistical Analyses

An iterative process was used to select the best fit model for explaining the variability between successive 1500 m time trials. Using the *lme4* package (Bates et al. 2015), two candidate linear models were developed for each physiological and performance variable; (1) fixed effect (time trial) only, (2) fixed effect (time trial) with a random effect (athlete). For each variable model fit, performed via the *MuMIn* package (Bartoń 2023), was substantially improved with the addition of the random effects term suggesting that the nested data structure required a mixed model approach. A visual check of model assumption plots, via the *performance* package (Lüdecke et al. 2021), revealed some evidence of heteroscedasticity. A logarithmic transformation was therefore applied to all variables before a final analysis with the mixed linear model log(variable ∼ time trial + (1|athlete)). As such, effects are reported as the percentage between‐trial difference. We have also reported marginal *R* ^2^ and ICC values to show the variance explained by our fixed and random effects, respectively. Missing values were explored using the *visdat* package (Tierney 2017) revealing missing data for heart rate (two observations), time trial expired gases (one observation) and ratings of perceived exertion (one observation). Linear mixed modelling can cope with missing values (Cnaan et al. 1997), so all participant data were included in the final analysis. Physiological and performance variables were plotted using raincloud plots via the *ggrain* package (Judd et al. 2023) that visualises raw data, probability density and key summary statistics, for example, median and mean (Allen et al. 2019; Patil 2021).

We have provided a visual assessment of statistical significance through the disposition of all fixed effect 95% confidence intervals (CI) against the null difference (i.e., 0%), but we have not interpreted these effects. Instead, to aid the practical interpretation of our data we compared the uncertainty of our fixed estimates against measurement error % coefficients of variation (CV) previously reported for all variables, that is, did the uncertainty of our between‐trial differences extend beyond that of measurement error. Measurement error values were: 3.3% for 1500 m time trial (Laursen et al. 2007), 12% for blood lactate (Stevens et al. 2015); 1.2% for mean and peak heart rate (Midgley et al. 2006); 3.3% for mean $\dot{V}$O_2_ (Stevens et al. 2015); 3.5% for peak $\dot{V}$O_2_ (Midgley et al. 2006); 2.4% for mean and peak RER (Midgley et al. 2006) 3.3% for running economy (Shaw et al. 2013); and, 2.2% and 2.6% for step length and step frequency (García‐Pinillos et al. 2022). Given that statistical equivalence can be obtained by visual inspection of the confidence interval in relation to the equivalence region (Lakens et al. 2018), we compared the 90% confidence interval (Dixon et al. 2018) calculated from our mixed linear models and plotted these against each variable's %CV, with evidence for statistical equivalence being non‐overlap of the upper and lower confidence intervals against the upper and lower bounds of the equivalence region (Lakens et al. 2018). Perceptual measures (sRPE, dRPE‐B, dRPE‐L) were analysed by ordinal regression via the *MASS* package (Venables and Ripley 2002) with models including a fixed effect for time trial and a random effect for athlete. All visualisations and analyses were performed in R (version 4.1.2, R Foundation for Statistical Computing).

## 3 Results

Data from baseline assessments, training and performance characteristics for the participants are provided in Table 1.

**TABLE 1:** Season's best performances in 800 and 1500 m distances, expressed as absolute (s) and relative to age‐group world record times (%), world athletics points, running experience, weekly running distance, lactate threshold, lactate turn point, maximal oxygen uptake and velocity at maximal oxygen uptake for the group, male and female participants. Data are presented as mean ± SD.

	Overall (*n* = 12)	Male (*n* = 10)	Female (*n* = 2)
800 m SB (s)	1:57.38 ± 0:07.76	1:54.00 ± 0:03.46	2:10.88 ± 0:01.22
800 m SB (%)	88.3 ± 2.6	88.7 ± 2.8	86.8 ± 0.4
1500 m SB (s)	4:04.18 ± 0:18.68	3:56.96 ± 0:09.94	4:36.62 ± 0:11.31
1500 m SB (%)	86.6 ± 3.8	87.2 ± 3.8	83.7 ± 3.4
World athletics points: 800 m	929 ± 86	917 ± 94	976 ± 20
World athletics points: 1500 m	901 ± 110	894 ± 119	931 ± 80
Running experience (*y*)	13 ± 8	14 ± 8	9 ± 2
Weekly distance (km)	85 ± 24	87 ± 24	57 ± 12
LT (km·h^−1^)	14.7 ± 1.1	15.0 ± 1.0	13.4 ± 0.9
LTP (km·h^−1^)	16.7 ± 1.1	16.9 ± 1.0	15.7 ± 1.2
v$\dot{V}$O_2_peak (km·h^−1^)	20.6 ± 1.3	21.0 ± 0.9	18.9 ± 1.8
$\dot{V}$O_2_peak (ml·kg·min^−1^)	58.3 ± 3.6	58.3 ± 4.1	58.3 ± 1.3

Abbreviations: LT, lactate threshold; LTP, lactate turn point; SB, season best performance; SD, standard deviation; $\dot{V}$O_2_peak, maximal oxygen uptake; v$\dot{V}$O_2_peak, velocity at maximal oxygen uptake.

### 3.1 Time Trial Performance

**FIGURE 1:**
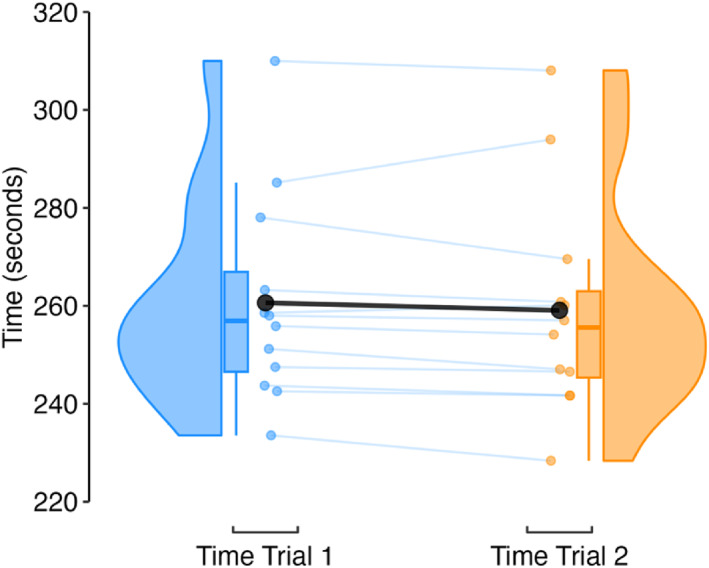
Raincloud plots and mean time trial 1500 m running time (black circle).

The fixed effect of time trial on 1500 m running time was −0.6% (95% CI −1.6, 0.3). A marginal *R* ^2^ of 0.002 and an ICC of 0.98 showed that random effect of athletes explained most the variance in running time (Figure 1).

### 3.2 Peak Physiological Responses

**FIGURE 2:**
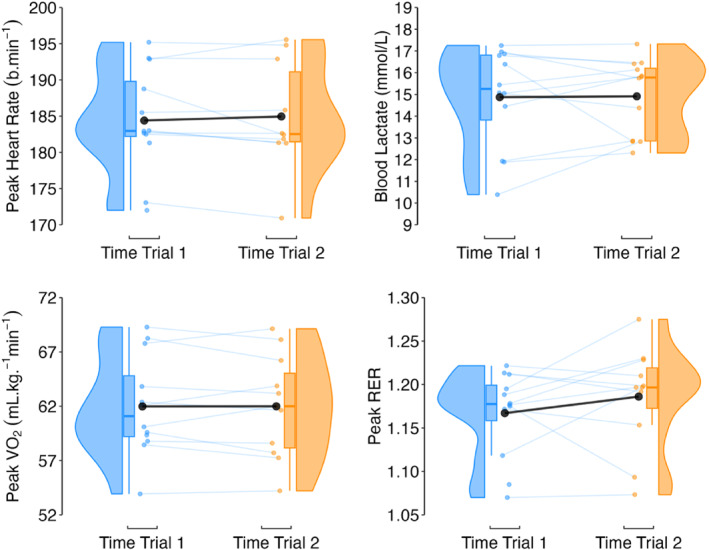
Raincloud plots and mean peak time trial response (black circle) for peak heart rate, blood lactate, peak $\dot{V}$O_2_ and peak RER. RER, respiratory exchange ratio; $\dot{V}$O_2_, oxygen uptake.

Fixed effects of time trial were: −0.5%; 95% CI −1.4, 0.4 (peak heart rate); −0.4%; 95% CI −1.8, 1.1 (peak $\dot{V}$O_2_); 0.8%; 95% CI −6.0, 8.1 (blood lactate concentration); and 1.2%; 95% CI −1.6, 4.1 (peak RER). Model variance was mostly accounted for by the athlete random effect, with ICC's ranging from 0.62 to 0.96 and low marginal *R* ^2^ (range 0.000–0.016) (Figure 2).

### 3.3 Mean Physiological Responses

**FIGURE 3:**
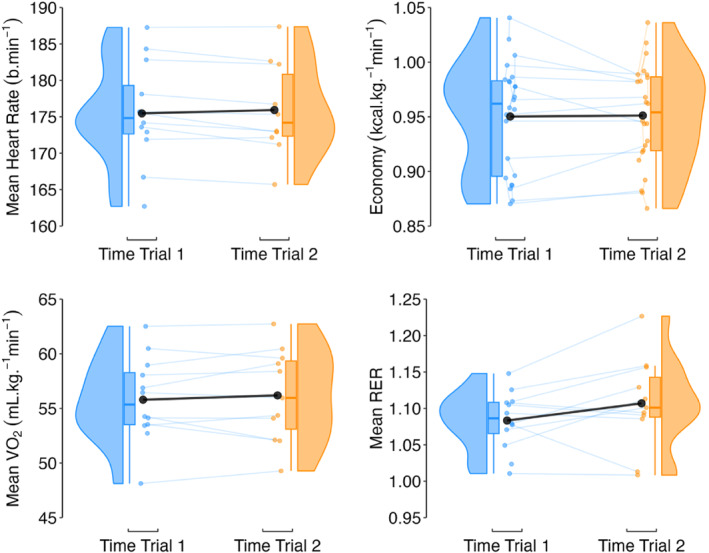
Raincloud plots and the mean time trial response (black circle) for mean heart rate, running economy, $\dot{V}$O_2_ and RER. RER, respiratory exchange ratio; $\dot{V}$O_2_, oxygen uptake.

Fixed effects of time trial were: −0.6%; 95% CI −1.0, −0.2 (mean heart rate); 0.1%; 95% CI −0.9, 1.1 (running economy); 0.2%; 95% CI −1.4, 1.8 (mean $\dot{V}$O_2_); and 1.8%; 95% CI −0.8, 4.3 (mean RER). Model variance was mostly explained by the athlete random effect with ICC's ranging from 0.68 to 0.99 and marginal R^2^s ranging from 0.000 to 0.033 (Figure 3).

### 3.4 Biomechanical Responses

**FIGURE 4:**
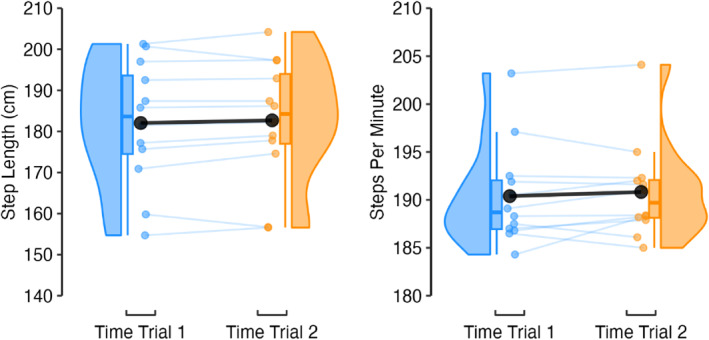
Raincloud plots and mean time trial response (black circle) for step length and step frequency.

Fixed effects of time trial were: 0.2%; 95% CI −0.3, 0.8 (step frequency); and 0.4%; 95% CI −0.4, 1.1 (step length). Model variance was mostly explained by the athlete random effect with ICC's of 0.99 and 0.94 and marginal R^2^s of 0.000 and 0.002, respectively (Figure 4).

### 3.5 Fixed Effects Interpretation

**FIGURE 5:**
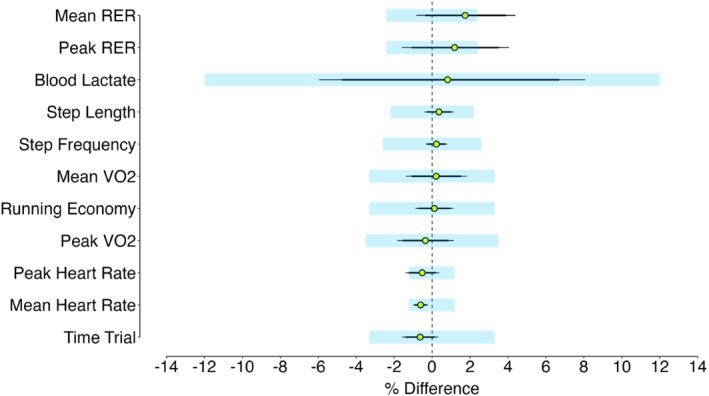
Between‐trial differences with 95% (thin line) and 90% (thick line) confidence intervals, expressed as %, for all performance, physiological and biomechanical variables. The previously reported % CVs for each respective variable are displayed via the light blue shaded area. CV, coefficient of variation; RER, respiratory exchange ratio; $\dot{V}$O_2_, oxygen uptake.

The uncertainty of our fixed physiological and performance estimates against the measurement error (%CV) previously reported for these variables is presented in Figure 5. With the exception for mean and peak RER, statistical equivalence was observed for all variables suggesting that uncertainty of these between‐trial differences are within measurement error.

### 3.6 Perceptual Measures

**FIGURE 6:**
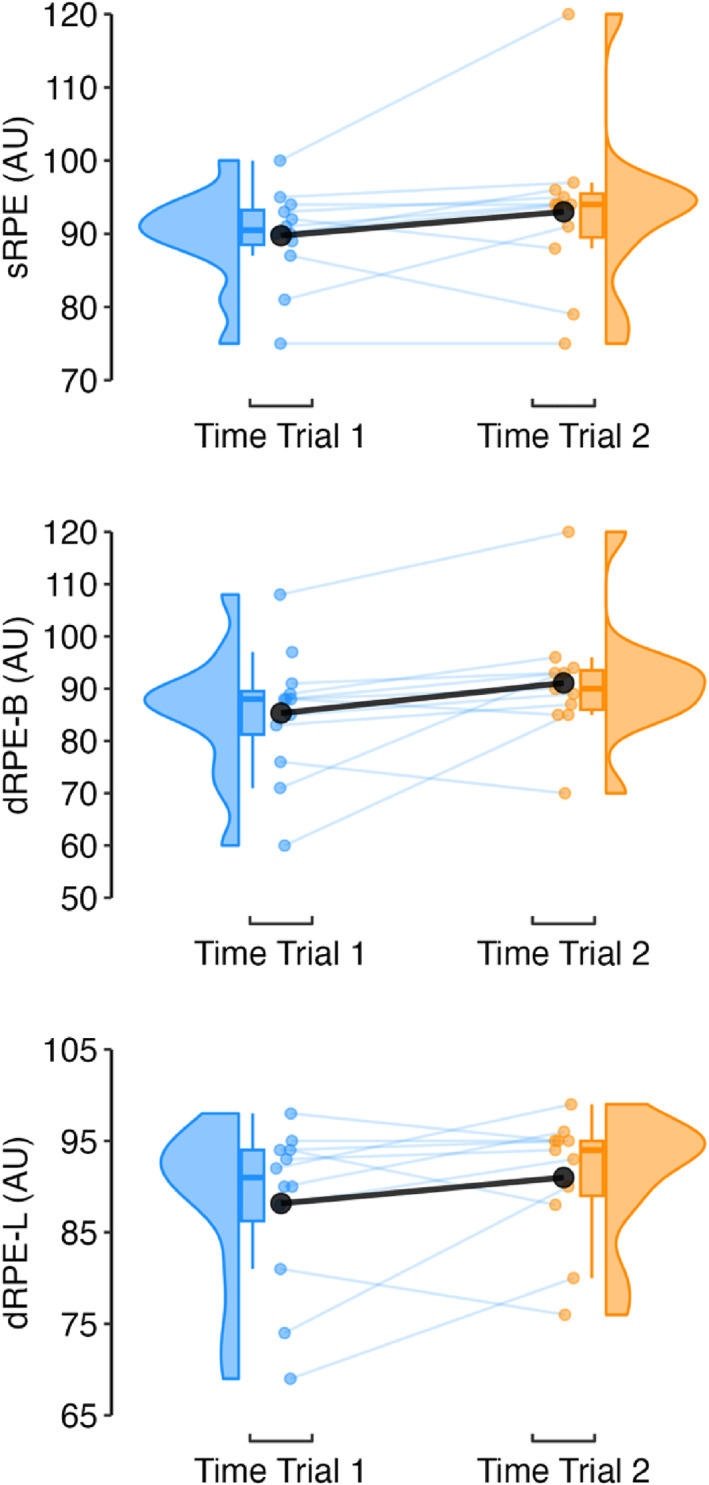
Raincloud and mean time trial response (black circle) for time trial sRPE, dRPE‐B and dRPE‐L. dRPE‐B, differential ratings of perceived exertion for breathlessness; dRPE‐L, differential ratings of perceived exertion for leg muscle exertion; sRPE, session ratings of perceived exertion.

**FIGURE 7:**
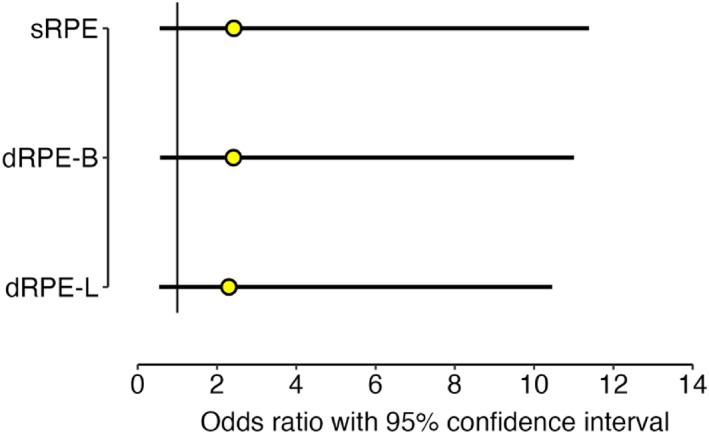
Odds ratios and 95% confidence interval for the fixed effect of time trial performance on all perceptual measures. dRPE‐B, differential ratings of perceived exertion for breathlessness; dRPE‐L, differential ratings of perceived exertion for leg muscle exertion; sRPE, session ratings of perceived exertion.

Perceptual data are presented in Figure 6. Athletes were ∼2.5 times more likely to rate exertion higher in the second time trial, but with considerable uncertainty surrounding each of these estimates (Figure 7).

## 4 Discussion

The aim of this study was to examine the impact of successive 1500 m self‐paced time trials in national‐standard middle‐distance specialists on performance and determinants of performance. National‐standard middle‐distance specialist runners appear to maintain 1500 m running performance on successive days. Peak and mean RER were greater for time trial 2, whilst athletes elicited similar responses for all other physiological measures as well as step frequency and length. Whilst mean speed was similar between time trials, athletes were ∼2.5 times more likely to rate exertion higher in time trial 2; however, there was considerable uncertainty surrounding the estimates. Without use of any structured recovery modalities, 24 h recovery permits National standard 1500 m specialists to maintain successive 1500 m time trial performance despite an increase in perceived exertion.

### 4.1 Performance Time

Time to complete two successive 1500 m time trials were similar when separated by 24 h in national‐standard middle‐distance specialists. Performing multiple race efforts within a day can impair subsequent performance in simulated cross‐country skiing (Stöggl et al. 2007) and in speed skating competitions (Konings and Hettinga 2018b). However, in speed skating World Cup events, race efforts on day one does not affect performance on day two in the 1500 m distances in male and female elite skaters (Konings and Hettinga 2018b) and in rowing, 24 h recovery is sufficient to maintain 2000 m rowing ergometer performance in collegiate standard female rowers (Hopkins et al. 2011). Therefore, despite differences in modality with regards to weight‐bearing versus non‐weight bearing, findings of the present study that a 24 h recovery period appears sufficient to maintain successive middle‐distance time‐trial performance appear similar to previous reports.

### 4.2 Physiological and Perceptual Responses

No impact of the prior time trial was observed for mean or peak heart rate, mean or peak $\dot{V}$O_2_, blood lactate concentration or running economy, although mean and peak RER were greater for time trial 2. Additionally, participants were more likely to rate higher perceived exertion for time trial two. Whilst mean and peak RER were not equivalent between trials, the increase for time trial two is small and when accompanied by equivalent blood lactate concentrations and mean and peak $\dot{V}$O_2_ values, is likely of no considerable physiological impact. No differences in mean HR and RPE have been reported between two 5 km time trials separated by 24 h, despite time trial two being performed 10 s slower than the initial trial (Bosak et al. 2009), although the distance of over three times the length of that of the present study should be considered. For the 1500 m event, $\dot{V}$O_2_peak (Ingham et al. 2008), v$\dot{V}$O_2_peak and running economy (Ingham et al. 2008; Bellinger et al. 2021) are key determinants of performance. Therefore, as performance was maintained between trials, it is not surprising that physiological responses remained unchanged. Many factors could contribute towards the maintenance of physiological function, such as the well‐trained status of these athletes or familiarisation with maximal efforts, as well as methodological factors such as difference in footwear compared with competition and time of season resulting in lower speed and forces. However, without comparisons contributing factors cannot be fully determined.

### 4.3 Biomechanical Demands

Step length and step frequency were equivalent between time trials. The present study employed a novel approach by allowing athletes to run at self‐selected speeds, reflecting competitive performance, while enabling continuous monitoring. Given that biomechanical outcomes are highly comparable between motorised treadmill and overground running (Van Hooren et al. 2020), self‐paced treadmill time trials offer a unique insight to running performance. It is, to the authors’ best knowledge, the first study to describe the impact of a prior 1500 m time trial on subsequent spatiotemporal characteristics, indicating that athletes maintain speed over successive trials by maintaining both step length and step frequency. It should be noted that the analysis was based on average data across the time trials and thus temporal changes within specific segments of the race (i.e., every 100m) and strategies may have been adopted. This requires further investigation, as well as a wider array of biomechanical variables; however, was beyond the scope of this investigation.

## 5 Conclusion

National‐standard middle‐distance specialists maintain performance, physiological function and spatiotemporal characteristics across successive 1500 m time trials separated by 24 h. These findings suggest that 24 h is a sufficient period to recover from a time‐trial without the use of structured recovery modalities. Further research should investigate the impact of tactical approaches (i.e., changes in pacing or not aiming to complete the distance as fast as possible) in earlier rounds on subsequent performance and consider replicating this study with different training groups, populations or in‐competition scenarios.

## 6 Limitations

Several limitations were present in this study. Motorised treadmill running might not fully reflect an overground 1500 m track race, but measuring ground reaction force outcomes over the entire distance was a key priority and participants were able to self‐select their pacing profile, which might somewhat offset these differences (Van Hooren et al. 2020). Athletes completed self‐paced individual laboratory‐based time trials in the absence of a direct head‐to‐head opponent. Performing with other competitors is shown to influence pacing behaviours and the regulation of exercise intensity (Konings and Hettinga 2018a; Crivoi do Carmo et al. 2022). Recreating real‐life competitive environments, such as the psychological pressures, emotions, motivations and excitement athletes experience surrounding competition remains difficult in laboratory‐based settings (Sanz et al. 2015). However, given the objective of the current study was to assess the effects of a prior effort on subsequent time trial performance, it is unlikely that the above limitations influenced this outcome. Race tactics differ between world championship medallists and world record performances (Casado et al. 2024), as medallists must achieve higher top speeds in the final part of the race to beat their opponents (Hanley et al. 2019). Therefore, further investigation in race scenarios is warranted. This study was performed early on in the season, not in the competition season and carbon plated shoes were included, which may have affected forces experienced by athletes and consequently responses to the trials. Additionally, we acknowledge that some of the variation surrounding differences were a result of inclusion of male and female participants. However, owing to the underrepresentation of female participants in sport science research, the inclusion of this data in the initial dataset and the high‐performance standard of these athletes (World athletics points, Table 1) we believe this is the best and most appropriate approach.

## Funding

The authors have nothing to report.

## Conflicts of Interest

The authors declare no conflicts of interest.
